# Supplementary material for: Association between oral microbiome and seven types of cancers in East Asian population: a two-sample Mendelian randomization analysis
Source: Front Mol Biosci. 2023 Nov 21;10:1327893. doi: 10.3389/fmolb.2023.1327893 (PMC10702768; doi:10.3389/fmolb.2023.1327893)
Supplement: Supplementary file 1 [file DataSheet1.ZIP › Supplementary Materials/MR plots of saliva/saliva═╝/ovarian cancer/pheno.3403_to_ovarian cancer_forest.pdf]

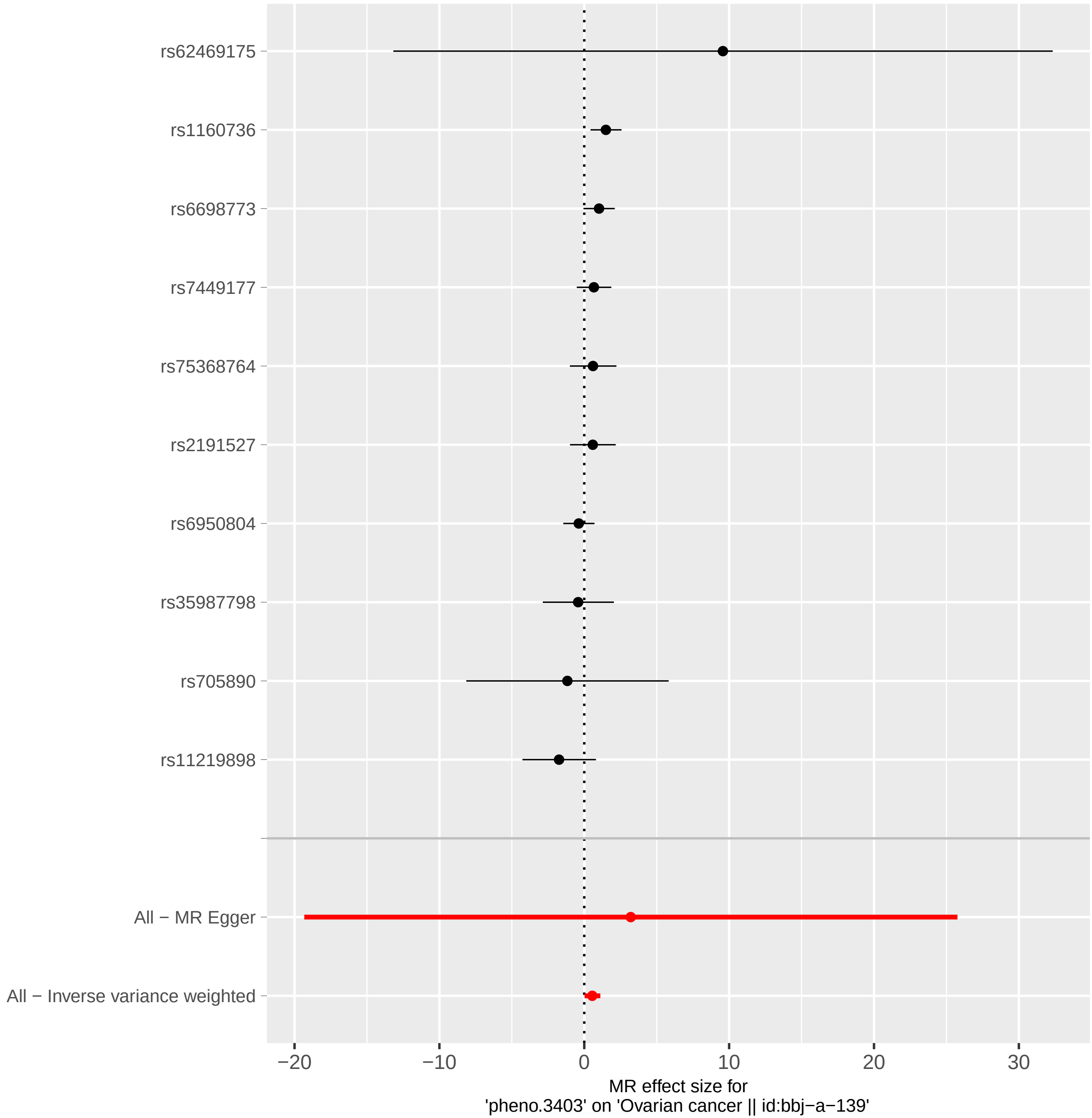

rs62469175
rs1160736
rs6698773
rs7449177
rs75368764
rs2191527
rs6950804
rs35987798
rs705890
rs11219898
All − MR Egger
All − Inverse variance weighted
−20
−10
0
10
20
30
MR effect size for
'pheno.3403' on 'Ovarian cancer || id:bbj−a−139'
